# Prevalence and factors associated with research misconduct among faculty in selected public universities in Eastern Uganda: a cross-sectional study

**DOI:** 10.1007/s44217-025-00945-1

**Published:** 2025-12-15

**Authors:** Emmanuel Tiyo Ayikobua, Ian G. Munabi, Sylvia Nabukenya, Ronald Opito, Erisa Sabakaki Mwaka

**Affiliations:** 1 Department of Physiology, School of Health Sciences, Soroti University, P.O. Box 211, Soroti, Uganda; 2 Department of Anatomy, School of Biomedical Sciences, Makerere University, College of Health Sciences, P.O. Box 7072, Kampala, Uganda; 3 Department of Public Health, School of Health Sciences, Soroti University, P.O. Box 211, Soroti, Uganda

**Keywords:** Research misconduct, Plagiarism, Fabrication, Falsification, Research ethics, Research integrity

## Abstract

We report the prevalence and associated factors of self-reported research misconduct (RM) among 70 faculty members across two recently established public universities in Eastern Uganda. A descriptive cross-sectional survey was conducted using a structured, anonymized self-administered questionnaire adapted from the Scientific Misconduct Questionnaire–Revised (SMQ-R). Data were analyzed using STATA version 16.0. Descriptive statistics summarized participant characteristics; bivariate and multivariable logistic regression were used to identify predictors of RM. The overall prevalence of self-reported RM, defined as engagement in any one of fabrication, falsification, or plagiarism (FFP), was 31.4% (22/70). Plagiarism and falsification were each reported by 20.0% of participants, while fabrication was reported by 1.4%. Most respondents were male (72.9%), held master’s-level qualifications (70.0%), and had received prior ethics training (81.4%). In multivariable analysis, prior ethics training (aOR = 0.27; 95% CI 0.07–0.98; p = 0.046) and the perception that RM is unacceptable (aOR = 0.27; 95% CI 0.07–0.98; p = 0.046) were independently associated with lower odds of self-reported RM. These findings indicate that RM is relatively common among faculty in Eastern Uganda. Although prior ethics training was associated with lower odds of self -reported RM, the persistence of misconduct suggests that existing ethics training programs need strengthening in both quality and institutional integration. Also, A multifaceted approach is needed to foster research integrity within academic institutions.

## Introduction

Research misconduct is defined as fabrication, falsification, or plagiarism (FFP) in the conduct, reporting, or review of research. It violates ethical and scientific norms [1]. Globally, the prevalence of self-reported misconduct ranges between 1.97 and 2.9%, but is believed to be higher in low-resource settings, including sub-Saharan Africa [2–4]. Fabrication (creating fictitious data) and falsification (manipulating research materials or results) have reported prevalences ranging from 4.7 to 22%, while plagiarism, the misappropriation of others’ ideas or work remains one of the most commonly reported offenses [5–7].

Such practices erode the credibility of science, waste public resources, and may harm research participants [8]. For instance, between 1991 and 2012, over $400 million in U.S. National Institutes of Health funding was linked to retracted research due to misconduct [9, 10]. These acts have had devastating consequences, including physical harm and career damage to both perpetrators and their institutions [11, 12].

Research misconduct is seldom acknowledged within universities let alone young institutions, hence the absence of effective strategies to address the issue [13]. In Uganda, efforts to develop a national policy on research misconduct are ongoing; however, no peer-reviewed studies have yet documented the prevalence and correlates of research misconduct among university faculty in the country. Most of the available African data come from Nigeria and Kenya, while Uganda has remained a “data-silent” setting despite being a rapidly growing hub for biomedical and public health research [14, 15].

This study is therefore the first empirical investigation of research misconduct among faculty in Eastern Uganda. Unlike previous work, it focuses specifically on newly established public universities, where research oversight systems are still developing and a lot of growth and competition to meet globally recognizable standards is underway. By documenting both prevalence and institutional/contextual correlates of misconduct, the study provides evidence directly relevant to Uganda’s emerging research integrity policies.

The study addresses the critical empirical evidence gap in Uganda through these three contributions: it provides national-level empirical data where none previously existed, it provides foundational data on research misconduct among faculty in newly established public universities, and it adds to the limited literature from low- and middle-income countries. It also contributes theoretically by applying Ajzen’s Theory of Planned Behavior to explain how training, attitudes, and institutional culture shape ethical decision-making among researchers.

This study aimed to determine the prevalence and facilitators of research misconduct among faculty members at selected public universities in eastern Uganda, to inform the policy development process.

### Theoretical framework basis

This study is guided by Ajzen's Theory of Planned Behavior (TPB), which posits that individual behavior is driven by behavioral intentions shaped by attitudes, perceived social norms, and perceived behavioral control [16]. In the context of research misconduct, TPB explores how researchers' personal beliefs, peer expectations, and institutional environments influence ethical decision-making [17, 18].

Key mediators such as ethics training, mentorship, perceived consequences of misconduct, and organizational justice also interact with the core constructs of TPB to shape [19–21]. This framework enables an examination of how both individual and institutional factors operate to either deter or facilitate engagement in misconduct.

We therefore hypothesize that prior formal training of research ethics is negatively associated with self-reported research misconduct. This could imply thatthe training enhances perceived behavioral control.

### Research misconduct: historical, conceptual, and epidemiological perspectives

Research misconduct (RM) has evolved from being considered a rare ethical anomaly to a recognized global concern with profound implications for the credibility of scientific knowledge [13]. Clear and consistent definitions of misconduct are fundamental to distinguishing unethical from acceptable research behavior. According to the U.S. Federal Policy on Research Misconduct (2002), RM is defined as a significant deviation from accepted research practices, committed intentionally, knowingly, or recklessly [22]. It encompasses three core forms: fabrication (making up data), falsification (manipulating processes or results), and plagiarism (using others’ work without attribution) [13, 23, 24]. While the U.S. emphasizes these three categories (commonly referred to as FFP), other jurisdictions vary. For instance, Nordic countries include serious deviations from broader ethical standards, while the U.K. definition extends to both intentional and unintentional violations [25–27]. This study adopts a broader interpretation aligned with the British approach, which better captures the range of behaviors that compromise research integrity.

The historical trajectory of RM reveals its deepening significance within academic and policy discourse. Early reports, notably by Stephen Lock in the 1970s, catalyzed attention to fraudulent research practices [27]. Prominent cases include William Summerlin's fabricated skin graft data and John Darsie's falsified findings at Harvard, which demonstrated that RM could occur even in elite institutions [27]. In the U.K., the Malcolm Pearce case in 1994 drew national attention when fabricated data in obstetric trials led to high-profile retractions and regulatory reforms [28]. Growing alarm over such incidents led to the first U.S. congressional hearings on RM in 1981, chaired by Albert Gore Jr., which uncovered misconduct in major research centers and underscored the lack of institutional mechanisms for accountability [29].

Despite increasing scrutiny, measuring the true extent of RM remains methodologically and ethically challenging. Definitions of misconduct vary, and not all problematic behaviors are consistently classified. Some frameworks exclude violations such as protocol deviations or failure to report misconduct [30], while others include these as critical components. Standard epidemiological constructs—such as point, period, or lifetime prevalence—are difficult to apply without precise population boundaries and time frames. Sampling entire research communities is often infeasible, and random selection is hindered by access restrictions and fear of reprisal [10, 31].

Nevertheless, available data suggest RM is more widespread than previously assumed. Fang et al. (2012) found that of 2047 retracted articles in PubMed-indexed journals from 1975 to 2012, 67.4% were due to misconduct, with 47% attributed to fabrication or falsification and 9.8% to plagiarism. Only 21.3% were withdrawn due to honest error [32]. Survey-based studies report even higher rates: a Middle Eastern study reported that 59.4% of researchers admitted to at least one act of misconduct, while 74.8% acknowledged knowing a colleague who had engaged in RM [3]. In Nigeria, self-reported prevalence among researchers was estimated at 68.9% [13].

Data from high-income countries also highlights the persistence of misconduct. A Dutch study reported fabrication and falsification rates of 4.3% and 4.2%, respectively—figures that, while seemingly low, are significant given the ethical implications [33]. Meanwhile, low- and middle-income countries (LMICs) remain underrepresented in RM literature, with scanty representations showing a high prevalence, despite being key sites for global health and clinical research [6, 10, 34, 35]. Ana et al. [37] highlighted significant structural deficits in LMICs for addressing RM, including a lack of institutional oversight, insufficient training, and weak enforcement systems [36, 37]. In Uganda specifically, there is a conspicuous absence of peer-reviewed studies estimating the prevalence of RM, particularly among academic faculty, making this an urgent area for inquiry and policy attention.

### Institutional integrity, faculty influence, and researcher awareness

Academic integrity is foundational to the credibility of higher education and scientific institutions. However, it is increasingly compromised by a spectrum of unethical practices, including academic misconduct (e.g., cheating, deception), research misconduct (FFP), and questionable research practices (QRPs) that blur ethical boundaries [38, 39]. These practices pose significant threats to research credibility and scholarly trust.

Faculty members play a central role in shaping ethical research environments, both through their conduct and their influence on students and peers. Research indicates that academic dishonesty often begins early in training and, if unaddressed, may persist in professional and research settings. For example, Brenda et al. (2018) found that a substantial proportion of students admitted to routinely manipulating laboratory results. Such behaviors are more prevalent in poorly supervised environments, emphasizing the need for early intervention and ethics education [40, 41].

Yet fostering a culture of integrity extends beyond institutional rules; it requires cultivating knowledge and awareness of research ethics. Knowledge in this context refers to the capacity to identify misconduct and understand ethical standards in research [42]. Limited awareness, especially among early-career researchers, is a key driver of misconduct. This is often compounded by pressures to publish, competition for funding, inadequate mentorship, and fragmented training systems [24, 43].

In low- and middle-income countries (LMICs), these challenges are amplified by systemic gaps in policy and governance. Uganda’s national research ethics guidelines, for instance, offer only minimal reference to integrity and lack operational definitions or enforcement mechanisms [44]. There is no dedicated institutional framework to investigate or prevent research misconduct, reflecting broader deficiencies in the country’s research oversight landscape [34, 45].

Conversely, institutions in high-income countries typically adopt comprehensive ethics policies, define misconduct clearly, and implement structured training programs. These have been shown to positively influence researcher behavior and promote ethical cultures [3]. However, challenges persist globally. Faculty members may feel reluctant to address misconduct due to discomfort, lack of institutional support, or fear of reprisal—resulting in underreporting and weak enforcement [3, 46]. In Eastern Uganda, empirical data on research misconduct are virtually absent. Yet studies from comparable contexts, including Nigeria, South Africa, and the Middle East, reveal high prevalence of RM and QRPs, though the underlying causes vary by region [6, 35]. This highlights the need for localized investigations that consider institutional culture, academic pressures, and policy environments.

This study addresses this critical gap by generating empirical data on the prevalence and correlates of research misconduct among faculty at public universities in Eastern Uganda. The findings aim to inform institutional reform, guide ethics training, and support the development of evidence-based policy tailored to low-resource academic environments.

## Materials and methods

### Study design and setting

A descriptive cross-sectional study was conducted between September 2023 and March 2024 at two newly established public universities in Eastern Uganda. For confidentiality, the institutions are anonymized and referred to as University A and University B.

### Study population and sampling

The target population comprised 86 faculty members with prior research experience, employed on permanent terms across four randomly selected faculties. Eligibility criteria included active involvement in research and provision of written informed consent. Faculty who declined to provide consent were excluded.

The required sample size was determined using the Krejcie and Morgan table for finite populations [47, 48]. From a total eligible population of 86 faculty members, the table recommended a minimum of 70 participants to achieve a representative sample with a 95% confidence level and a margin of error of ± 5%. This calculation supports the adequacy of our sample size for the study’s objectives. While larger samples would allow for more precise estimates and stronger statistical power, the achieved sample was sufficient to detect meaningful associations within the defined population.

### Data collection

Data was collected using a structured online survey tool that was adapted from the modified scientific Misconduct Questionnaire-Revised (SMQ-R) and modified [10, 15, 35, 49]. The tool comprised questions on demographic information, prior research experience, student supervision, and prior ethics training of participants, work-related and publication pressures, responsible mentorship, and social factors such as personal beliefs, normative practices, and organizational justice. Participants were asked for their level of agreement using a four-point Likert scale, ranging from strongly agree to strongly disagree. To establish the prevalence of research misconduct, two questions were asked on fabrication, four on plagiarism, and six on falsification. A participant who admitted having committed any one of falsification, plagiarism, or fabrication was considered a count contributing to the prevalence of research misconduct.

### Data management and analysis

Data was cleaned and exported to Stata version 15.0, a statistical software for data analysis. The dependent variable was self-reported research misconduct, which was a composite of fabrication, falsification, and plagiarism [1]. Any participant who admitted to any act of FFP was considered to have engaged in research misconduct. Participants who admitted to having engaged in more than one act of research misconduct were still counted as a “Yes” count in research misconduct.

The independent variables included socio-demographics, prior ethics training, research experience, academic positions, academic rank, and college of affiliation. The facilitators and deterrents of research misconduct were categorized in Table 1.

**Table 1:** Showing the Facilitators of research misconduct on a Likert scale arrangement

Variable	Strongly agree n (%)	Agree N (%)	Disagree N (%)	Strongly disagree n (%)
I am given more work than I can accomplish	39 (55.7)	29 (41.4)	2 (2.9)	0 (0.0)
My academic career depends on research funding	17 (24.3)	28 (40.0)	12 (17.1)	13 (18.6)
Research collaboration is common among researchers in the same field	6 (8.6)	40 (57.1)	17 (24.3)	7 (10.0)
Researchers in my field are united	9 (12.9)	40 (57.1)	15 (21.4)	6 (8.57)
All research data should be declared	61 (87.1)	8 (11.4)	1 (1.4)	0 (0.0)
Researchers get away with research misconduct	6 (8.6)	28 (40.0)	17 (24.3)	19 (27.1)
Research misconduct is respectable and praiseworthy	2 (2.6)	11 (15.7)	23 (32.9)	34 (48.6)
Research misconduct is not a big deal	4 (5.7)	16 (22.9)	18 (25.7)	32 (45.7)
Researchers work on too many protocols	19 (27.1)	28 (40.0)	21 (30.0)	2 (2.9)
Researchers prefer to take shortcuts	5 (7.1)	15 (21.4)	27 (38.6)	23 (32.9)
Resource and task allocation in my department is fair	7 (10.0)	21 (30.0)	21 (30.0)	21 (30.0)
Decisions about promotions in my department are reasonable	23 (32.9)	21 (30.0)	14 (20.0)	12 (17.1)
There is insufficient censure of research misconduct by the university	11 (15.7)	17 (24.3)	25 (35.7)	17 (24.3)
Low likelihood of detecting research misconduct by peer review process	8 (11.4)	25 (35.7)	16 (22.9)	21 (30.0)
No policy on research misconduct	13 (18.6)	14 (20.0)	18 (25.7)	25 (35.7)
Mentor offers guidance on ethical research practice and publication	13 (18.6)	14 (20.0)	31 (44.3)	12 (17.1)

“Likert responses are presented descriptively in Table 1 while for the regression analysis, the Likert-type data scale was dichotomized to a binary output scale for all independent variables for simplicity in analysis focusing on extremes and to ensure consistency and transparency between descriptive and inferential analyses. The responses "strongly agree” and "agree”, were all considered as “Agree”, and “disagree” and “strongly disagree” were considered as “Disagree”. Therefore, the final output was binary with either “Agree” or “Disagree” [6, 10].

Analysis was done at three different levels univariate, bivariate, and multivariable. Descriptive statistics were used at univariate level. Continuous variables were summarized as frequencies and inter-quartile ranges (IQR). At the bivariate level, logistic regression analysis was conducted to assess the relationship between the research misconduct and the independent variables already described above. Given the limited faculty population (N = 86), our sample size (n = 70) constrained the statistical power for multivariable analyses. To reduce the risk of excluding potentially meaningful predictors, we adopted a p-value threshold of < 0.1 at the bivariate stage, as previously applied in studies with small samples. This approach allowed a more inclusive selection of candidate variables while maintaining interpretability [6].

## Results

### Socio-demographics

Seventy individuals participated in the study, with equal representation of both universities. Most participants were male (72.9%, n = 51), master degree holders (70%, n = 49) and had the academic rank of lecturer, (71.4%, n = 50). Thirty participants (42.9%) studied abroad, 57 (81.4%) had prior research ethics training, and a greater majority (88.6%) had conducted research in the past 3 years (Table 2).

**Table 2:** Showing the socio-demographic characteristics of participants

Number of publications	Frequency	Percentage
	3.5(IQR: 1–10)	
Sex		
Female	19	27.1
Male	51	72.9
University of affiliation		
University A	35	50.0
University B	35	50.0
Highest qualification attained		
Bachelors	10	14.3
Masters	49	70.0
PhD	11	15.7
Academic position		
Tutorial assistant	9	12.9
Lecturer	50	71.4
Senior lecturer and above*	11	15.7
Ever studied abroad**		
No	40	57.1
Yes	30	42.9
Ethics training		
No	13	18.6
Yes	57	81.4
Research field		
Biomedical and life sciences	48	68.6
Engineering	16	22.9
Social and behavioral sciences	6	8.6
School/Faculty		
Agricultural and animal science	15	21.4
Engineering and technology	7	10.0
Health sciences	44	62.9
Science and education	4	5.7
Did research the past 3 years		
No	8	11.4
Yes	62	88.6

Senior Lecturer and above* = Senior Lecturer, Associate Professor, Professor

Ever studies Abroad** = Attained academic qualification from any country other than Uganda

Most participants expressed concern about the workload, with 97.1% *agreeing* they were *given more work than they could accomplish*. Notably, 98.4% of participants either *strongly agreed or agreed* that *all research data should be declared*. However, almost 48.6% of participants agreed that *researchers get away with research misconduct*, while only 51.4% *disagreed*. Perceptions regarding the acceptability of research misconduct were generally negative; 81,5% either disagreed or strongly disagreed that *research misconduct is respectable or praiseworthy*, while 71.4% *disagreed* that misconduct is *"not a big deal".* Regarding the facilitators of research misconduct, participants were non-committal, with 40% agreeing that institutions do not condemn research misconduct and 60% disagreeing. The same was true for the likelihood of detecting research misconduct my peer review systems. About 61% disagreed that the lack of policies was a facilitator of research misconduct.

### Prevalence of research misconduct

About 31.4% of participants reported involvement in plagiarism, falsification or fabrication as shown in Table 3. Plagiarism and falsification were the most common forms of self-reported research misconduct at 20% each.

**Table 3:** Prevalence of research misconduct among faculty at public universities in Eastern Uganda

	Yes Freq (%)	No Freq (%)	Unsure Freq (%)	Prevalence n (%)
Fabrication				1 (1.4)
Have you ever fabricated data?	1 (1.4)	63 (90.0)	6 (8.6)	
Have you made up data for research that did not exist?	0 (0.0)	65 (92.9)	5 (7.1)	
Plagiarism				14 (20.0)
Have you ever plagiarized data?	4 (5.7)	57 (81.4)	9 (12.9)	
Have you used phrases or ideas of others without their permission?	9 (12.9)	44 (62.9)	17 (24.3)	
Have you used another person’s article and not cited them	2 (2.9)	58 (82.7)	10 (14.3)	
Copied in whole a text from another person’s work and called it my own	6 (8.6)	60 (85.7)	4 (5.7)	
Falsification				14 (20.0)
Have you ever falsified data?	1 (1.4)	69 (98.6)	0 (0.0)	
Have you misleadingly presented results?	1 (1.4)	60 (85.7)	9 (12.9)	
Have you changed data before or after performing data analysis?	7 (10.0)	59 (84.3)	4 (5.7)	
Have you concealed results that contradicted previous research you published?	2 (2.9)	66 (94.3)	2 (2.9)	
Have you modified the results of a study under pressure from the funder	1 (01.4)	68 (97.1)	1 (1.4)	
Decided whether to exclude data after looking at the impact of doing so on the results?	6 (8.6)	59 (84.3)	5 (7.1)	
Self-reported Research misconduct				22 (31.4)

### Awareness of colleagues involved in research misconduct among

When asked whether they knew colleagues who were involved in any form of research misconduct, 72.9% (n = 51) responded in the affirmative. Forty (57.1%) knew someone who had engaged in fabrication, 68.6% in plagiarism, and 58.6% in falsification (Table 4).

**Table 4:** Awareness of a colleague involved in research misconduct

	Response	Freq (%)
Fabrication		
Are you aware of a colleague who fabricated data?	Yes	40 (57.1)
	No	25 (35.7)
	Unsure	5 (7.7)
Plagiarism		
Are you aware of anyone who has plagiarized data?	Yes	48 (68.6)
	No	19 (27.1)
	Unsure	3 (4.3)
Falsification		
Are you aware of anyone who has falsified data?	Yes	41 (58.6)
	No	24 (34.3)
	Unsure	5 (7.1)
Total		51 (72.9)

### Facilitators of research misconduct

Table 5 shows the association between research misconduct and the facilitators of research misconduct. At bivariate analysis, unreasonable departmental decisions on promotion (OR 4.5, CI 1.31–15.28, p = 0.016) was the only facilitator of research misconduct as shown in Table 5. Importantly, participants with research ethics training were less likely to be involved in research misconduct p = 0.061 (OR 0.31, CI 0.09–1.06, p = 0.06) although the difference was not significant.

**Table 5:** Association between facilitators and research misconduct

Variable		n = 70	Research misconduct N (%)		Odds ratio	P-value
			Yes	No	COR (95% CI)	
Number of students supervised			3 (IQR: 1–6)			
Number of publications			3.5 (IQR: 1–10)			
Sex	Female	19	8 (42.1)	11 (57.9)	1	
	Male	51	14 (27.4)	37 (72.6)	0.52 (0.17–1.56)	0.244
University	University A	35	13 (37.1)	22 (62.9)	1	
	University B	35	9 (25.7)	26 (74.3)	0.59 (0.21–1.62)	0.305
Academic rank	Bachelors	10	4 (40.0)	6 (60.0)	1	
	Masters	49	17 (34.7)	32 (65.3)	0.80 (0.20–3.22)	0.750
	PhD	11	1 (9.1)	10 (90.9)	0.15 (0.01–1.67)	0.123
Academic position	Tutorial Assistant	9	4 (44.4)	5 (55.6)	1	
	Lecturer	50	14 (28.0)	36 (72.0)	0.49 (0.11–2.08)	0.330
	Sen. Lec/above*	11	4 (36.4)	7 (63.6)	0.71 (0.12–4.32)	0.714
Research field	Biomedical/life sciences	48	15 (31.2)	33 (68.8)	1	
	Eng/Nat/soc/behav/sci	22	7 (31.8)	15 (68.2)	1.03 (0.35–3.03)	0.962
Ever studied abroad*	No	40	15 (37.5)	25 (62.5)	1	
	Yes	30	7 (23.3)	23 (76.7)	0.51 (0.18–1.47)	0.210
Did research the past 3 years	No	8	2 (25.0)	6 (75.0)	1	
	Yes	62	20 (323)	42 (67.7)	1.43 (0.27–7.72)	0.679
Ethics training	No	13	7 (53.9)	6 (46.1)	1	
	Yes	57	15 (26.3)	42 (73.7)	0.31 (0.09–1.06)	0.061*
College of Affiliation	Health Sciences	44	13 (29.5)	31 (70.5)	1	
	Others*	26	9 (34.6)	17 (65.4)	1.23 (0.45–3.56)	0.659
I am given more work than I can accomplish	Agree	68	22 (32.4)	46 (67.7)		#
	Disagree	2	0 (0.0)	2 (100.0)		
My academic career depends on research funding	Agree	59	20 (33.9)	39 (66.1)	1	
	Disagree	11	2 (18.2)	9 (81.8)	0.43 (0.085–2.20)	0.313
Research collaboration is common among researchers in the same field	Agree	46	16 (34.8)	30 (65.2)	1	
	Disagree	24	6 (25.0)	18 (75.0)	0.63 (0.21–1.89)	0.405
Researchers in my field are united	Agree	49	18 (36.7)	31 (63.3)	1	
	Disagree	21	4 (19.1)	17 (81.0)	0.41 (0.12–1.39)	0.151
All research data should be declared	Agree	69	21 (30.4)	48 (69.6)		
	Disagree	1	1 (100.0)	0 (0.0)		#
Researchers get away with research misconduct	Agree	34	11 (32.4)	23 (67.7)	1	
	Disagree	36	11 (30.6)	25 (69.4)	0.92 (0.34–2.52)	0.871
Research misconduct is respectable and praiseworthy	Agree	13	7 (53.9)	6 (46.2)	1	
	Disagree	57	15 (26.3)	42 (73.7)	0.31 (0.09–1.06)	0.061*
Research mis conduct is not a big deal	Agree	20	9 (45.0)	11 (55.0)	1	
	Disagree	50	13 (26.0)	37 (74.0)	0.43 (0.15–1.27)	0.126
Researchers work on too many Protocols	Agree	47	18 (38.3)	29 (61.7)	1	
	Disagree	23	4(17.4)	19 (82.6)	0.34 (0.10–1.16)	0.084*
Researchers prefer to take shortcuts	Agree	30	13 (43.3)	17 (56.7)	1	
	Disagree	40	9 (22.5)	31 (77.5)	0.38 (0.13–1.07)	0.067*
Resource and task allocation in my department is fair	Agree	21	5 (23.8)	16 (76.2)	1	
	Disagree	49	17 (34.7)	32 (65.3)	1.7 (0.53–5.44)	0.372
Decisions about promotions in my department are reasonable	Agree	28	4 (14.3)	24 (85.7)	1	
	Disagree	42	18 (42.9)	24 (57.1)	4.5 (1.31–15.28)	0.016*
There is insufficient censure of research misconduct by the university	Agree	36	12 (33.3)	24 (66.7)	1	
	Disagree	34	10 (29.4)	24 (70.6)	0.83 (0.30–2.29)	0.724
Low likelihood of detecting research misconduct by peer review process	Agree	33	13 (39.4)	20 (60.6)	1	
	Disagree	37	9 (24.3)	28 (75.7)	0.49 (1.77–1.38)	0.178
No policy on research misconduct	Agree	27	10 (37.0)	17 (63.0)	1	
	Disagree	43	12 (27.9)	31 (72.1)	0.66 (0.23–1.84)	0.424
Mentor offers guidance on ethical research practice and publication	Agree	48	12 (25.0)	36 (75.0)	1	
	Disagree	22	10 (45.5)	12 (54.6)	2.5 (0.86–7.24)	0.091*

Others = agriculture and animal resources, science and education, Engineering and technology

Master’s and above = have Master’s degree and PhD

Sen. Lec/above = Senior Lecturer, Associate Professor, Professor

Eng/Nat/soc/behav/scie = Engineering/Natura/social/behavioral sciences

Ever studied Abroad = attained any of the degrees or certificates from any country other than Uganda

^#^omitted values because the variable did not meet the terms for logistic regression

*statistically significant value. To be exported for multivariable analysis

Variables with p ≤ 0.1 were then entered in a multivariable model (Table 6). Participants with ethics training (aOR 0.27, 95% CI 0.07–0.98, P = 0.046) and those who disagreed that "research misconduct is respectable and praiseworthy” were significantly less likely to report involvement in research misconduct.

**Table 6:** Multi-variate analysis logistic regression model

Variable	Response	Unadjusted Odds ratio (95% CI)	p-value	Adjusted Odds ratio	p-value
Prior ethics training	No	1			
	Yes	0.31 (0.09–1.06)	0.061*	0.27 (0.07–0.98)	0.046*
Research misconduct is respectable and praiseworthy	Agree	1			
	Disagree	0.31 (0.09–1.06)	0.061*	0.27 (0.07–0.98)	0.046*
Research misconduct is not a big deal	Agree	1			
	Disagree	0.34 (0.10–1.16)	0.084*		
Researchers prefer to take shortcuts	Agree	1			
	Disagree	0.38 (0.13–1.07)	0.067*		
Resource and task allocation in my department is fair	Agree	1			
	Disagree	4.5 (1.31–15.28)	0.016*		
Mentor offers guidance on ethical research practice and publication	Agree	1			
	Disagree	2.5 (0.86–7.24)	0.091*		

*Statistically significant values

## Discussion

This study determined the prevalence and determinants of research misconduct among faculty members at two Public Universities in Eastern Uganda. The overall prevalence of committing at least one form of research misconduct was 31.4%, with plagiarism and falsification being the most prevalent.

Earlier studies in Kenya reported a similar prevalence of self-reported research misconduct at 35.8%, with self-reported plagiarism and falsification at 22% [31]. They, however, reported a higher prevalence of self-reported data fabrication at 24% compared to just 1% in our study.

The prevalence of self-reported research misconduct in other low and middle-income countries (LMICs) is higher than in our study, ranging from 42% in20 Nigeria to 59% in the Middle East (17, 21). In comparison to high-income countries (HICs), the prevalence of self-reported research misconduct is estimated at below 5% [3, 50]. The difference in the prevalence of research misconduct between LMICs and HICs may be attributed to the absence of regulatory frameworks and weak institutional oversight. The situation may also be exacerbated by pressures to publish, limited funding, and career advancement needs that are disproportionately predominant among LMICs [3, 50, 51]. Therefore, this calls for regular sensitization and robust regulatory structures and processes, especially in LMICs.

Our findings reported that plagiarism is the most prevalent self-reported form of misconduct. This was similar to a study in Kenya (22%), although our prevalence was much higher than that reported by earlier studies in Nigeria (9% and 4.5%), who attributed their low prevalence to poor awareness of research ethics [10, 13, 52]. To explain our findings, literature denotes plagiarism as a less severe form of misconduct because it involves the appropriation of others' work without altering the facts in the data [7]. As such, many researchers may intentionally or unintentionally commit plagiarism without fear of reprimand [53, 54]. However, from an ethical standpoint, it still undermines academic integrity and can have significant consequences for the individual researcher and their institution [13]. Conversely, falsification is perceived to present a more severe ethical breach, as it involves the deliberate manipulation of data to achieve desired outcomes, not necessarily facts [55]. In our study, falsification was reported by 20% of participants. Falsification is less likely to be detected by any software applications, participants are more inclined to engage in it, and not worry of apprehension [54]. The high prevalence of falsification in our study raises concerns, particularly given its potential impact on public health and safety. A classic example of this occurred during the COVID-19 pandemic, when falsified data on treatments like hydroxychloroquine led to inappropriate medical practices [56].

The prevalence of self-reported data fabrication was 1%. In stark contrast, a Kenyan study 24% of respondents reported fabrication [31]. This disparity may reflect participants' reluctance to disclose acts of fabrication due to the severe ethical implications of creating false datasets. This is supported by findings from a meta-analysis that showed that while 10% of participants expressed a willingness to engage in fabrication to expedite publication, only 0.8% actually reported doing so, indicating a tendency for underreporting [3]. Data fabrication is universally condemned on moral, ethical, cultural, and scientific grounds and this likely contributes to self-underreporting as culprits fear self-incrimination [57].

Participants with prior ethics training were less likely to self-report engaging in research misconduct. This resonates with earlier studies in the Middle East that stated that research ethics training enhances awareness and understanding of issues surrounding research integrity, potentially reducing misconduct [6, 52]. Efforts to boost research ethics in Uganda have been made by increasing short and long-term courses within Uganda. These efforts have yielded results as observed in our study, where 81% of participants had prior ethics training and 81.4% considered research misconduct unacceptable. Individuals with ethics training have favorable attitudes toward research ethics [14, 18]. Also, the study by Felaefel et al. in the Middle East reported a significant association between prior research experience and researcher attitudes [6]. We therefore posit that when done correctly, ethics training plays a crucial role in fostering responsible conduct of research by increasing awareness, shaping attitudes and reinforcing ethical research-related norms. To maximize this effect, however, the training must be contextually relevant, off high quality, and integrated into routine supportive institutional networks.

The perception of institutional leniency towards research misconduct exacerbates the problem. Over half of the participants believed that researchers frequently escape consequences for unethical behavior. This is compounded by 36% of participants who reported insufficient institutional penalties for misconduct, and an equal percentage that there are low detection rates of unethical practices in their institutions. These factors create an environment where researchers may feel emboldened to engage in misconduct, despite having received ethics training [58].

Interestingly, our study found that participants were more likely to report observing colleagues engaged in misconduct than admitting to their own involvement, and this is consistent with the literature [2, 6, 31]. These studies may be attributed to the social stigma surrounding research misconduct, which could lead to dishonesty in self-reporting, especially in studies without mechanisms to incentivize truthful responses [59]. Moreover, it remains unclear whether reported observations involved distinct incidents or the same cases reported by multiple individuals. The inherent difficulty in detecting research misconduct could also result in incorrect or exaggerated observations [3].

In conclusion, this study contributes to emerging evidence on research misconduct in LMICs by revealing that nearly one-third of faculty in Eastern Uganda reported involvement in at least one act of misconduct. Unlike studies from high-income settings, where prevalence is < 5%, and from Nigeria/Kenya, where prevalence is higher but drawn largely from medical researchers, our findings capture faculty across diverse disciplines in newly established universities. This highlights the challenges of research oversight in rapidly expanding higher education systems. The findings highlight that misconduct is not solely a consequence of individual failings but also a broader systemic, institutional, and contextual influence. While the study associated ethics training with positive attitudes and lower self-reported misconduct, its effectiveness depends on quality, relevance, and integration within supportive institutional environments. Strengthening research integrity, therefore, requires a multi-pronged approach that combines education with institutional accountability.these findings directly inform Ugandas ongoing efforsts to draft national guidelines on research integrity.

### Limitations and strengths of the study

The study presented several limitations. First, the survey was based on self-reports of research misconduct, which is considered an incriminating and stigmatizing behavior. It is therefore most often characterized by underreporting incidents. Besides the dishonesty in reporting, challenges of recall bias are also common. This implies that research misconduct could be a bigger problem than reported. Secondly, the comparability of our study with other statistics was limited by taxonomy differences in the definition of research misconduct. Our definition from Nicholas H. Steneck [1] did not include questionable research practices. In our inferencing, we used statistics that adopted a similar taxonomy [13]. Third, The sample size was constrained by the actual number of eligible faculty, which reduced statistical power, especially for multivariable regression. Although we adopted a p-value threshold of < 0.1 at the bivariate stage to capture potential associations, the small sample remains a limitation. Future research with larger cohorts across multiple universities is recommended to validate and extend these findings [6]. Finally, the cross-sectional design does not allow for establishing causality between variables, and the list of misconduct facilitators studied may not be exhaustive.

## Data Availability

The datasets generated and/or analyzed during the current study are not publicly available due to ethical restrictions but are available from the corresponding author upon reasonable request. Data are stored securely and in compliance with institutional and ethical guidelines.
